# Regulating short-form video addiction: a three-tiered legal framework for the protection of Chinese minors

**DOI:** 10.3389/fpubh.2026.1848304

**Published:** 2026-07-13

**Authors:** Xia Wang, Jennifer S. Stevenson, Jiayi Wang

**Affiliations:** 1 School of Law, Beijing Jiaotong University, Beijing, China; 2 School of Law, Willamette University, Salem, OR, United States; 3 School of Law, China University of Political Science and Law, Beijing, China

**Keywords:** adolescent mental health, algorithmic governance, digital public health, addictive design, minor protection, platform regulation, short-form video addiction, three-tiered prevention

## 1 Introduction

Short-form video addiction (SFVA) among Chinese minors represents an engineered public health challenge. Personalized algorithms on short-form video platforms (SFVPs) effectively exploit the neuropsychological vulnerabilities of minors, thereby leading to disproportionate and long-lasting harm to their mental health and development. Although the *Law on Protection of Minors* (1) and *Internet Information Service Algorithmic Recommendation Management Provisions* (2) acknowledge the issue, the absence of clear and enforceable definitions for key terms such as “addictive design” has created a persistent implementation gap. Drawing on the public health model of three-tiered prevention, we propose a comprehensive legal framework. This layered approach translates vague prevention principles into concrete and enforceable governance measures for platform regulation. Our proposal engages with ongoing scholarly debates in legal and political science on digital platform governance, particularly the balance between state intervention, platform self-regulation, and family/school empowerment, while offering a focused interdisciplinary perspective through a public health prevention lens.

## 2 The design-induced addiction and harms

SFVA among minors is not accidental, but arises primarily from deliberately designed platform features and algorithmic mechanisms optimized to maximize user engagement.

Engaging with SFVPs activates the nucleus accumbens, triggering dopamine release and generating intense pleasure. When a video ends, dopamine levels decrease rapidly, creating a transient discomfort that drives users to keep swiping (3, 4). Moreover, the prefrontal cortex, which governs impulse control, decision-making, and risk assessment, continues to mature into the early 20s. This developmental stage makes adolescents particularly susceptible to immediate and intermittent rewards (5). These neuropsychological mechanisms explain why certain platform design features can become powerfully addictive for minors.

### 2.1 Platform design features as drivers of addictive engagement

Chinese SFVPs have developed highly sophisticated systems in which core interfaces and functional features are optimized to maximize user time spent and engagement frequency.

Infinite scroll eliminates natural stopping cues by enabling nonstop consumption, often requiring users only to swipe upward. Unlike traditional media with clear endings or structured breaks, this design creates an endless stream that encourages prolonged and habitual engagement (6, 7). Platforms further sustain prolonged usage through reward-based incentive mechanisms and immediate sensory feedback, such as likes, comments, and notifications. These features activate variable reinforcement schedules that encourage repeated checking and extended engagement sessions (8).

Engagement-maximizing algorithms prioritize content that evokes strong emotions, such as surprise, anger, or curiosity, thereby driving faster and more frequent user interactions. Platforms continuously refine addictive design features through the analysis of large-scale user behavioral data, creating self-reinforcing feedback loops that optimize user engagement (6). In addition, information cocooning, whereby AI-driven recommendations reinforce users' existing preferences and create homogeneous information environments, can further narrow information consumption patterns (9). For minors, even brief exposure to extreme or toxic content can trigger rapid algorithmic amplification, contributing to digital echo chambers that adversely affect their mental health and social wellbeing (10).

### 2.2 Documented harms to minor development

Short-form video consumption is associated with significant cognitive impairments. A meta-analysis of 71 studies involving 98,299 participants found that increased short-form video use was linked to “poorer cognition” (11). This association manifests as difficulties in sustained attention and impulse control. Additionally, SFVA is closely associated with impaired emotional regulation. Many minors rely on SFVPs for instant gratification and emotional escape, which may hinder the development of healthy coping skills and make it harder to manage everyday stressors. A longitudinal study of 1,143 Chinese high school students further revealed a bidirectional relationship between anxiety symptoms and SFVA, with impaired delay of gratification acting as a key mediator (12).

Excessive engagement with SFVPs is also linked to declines in social functioning (13). When minors have less time for face-to-face interactions, they are more likely to avoid social situations, communicate less with family members, and feel increasingly estranged from their peers. Moreover, recommendation algorithms can distort value formation among minors by promoting materialistic values, encouraging consumerism, and contributing to the fragmentation of cognitive structures (14).

## 3 Limitations of China's current regulatory framework

China's current regulatory framework remains insufficient to effectively address platform-induced SFVA among minors. This shortfall stems from three key limitations.

### 3.1 Absence of operational legal definitions

A key limitation is the absence of a clear and operational legal definition of “addictive design.” While existing legislation addresses addiction-related issues, it fails to specify which specific design elements are subject to regulation. As a result, platforms lack clear ex ante guidance on high-risk features. In addition, the diagnosis of digital addiction remains challenging due to the lack of universally accepted diagnostic criteria, particularly regarding the necessary combination of loss of control, psychological dependence, and functional impairment (3).

### 3.2 Ambiguity in regulatory responsibility

The ecosystem surrounding SFVA includes multiple stakeholders: platform operators, algorithm developers, content creators, users, and importantly, families and schools, which exercise direct supervisory authority over minors' digital behavior. However, China's legal system does not clearly allocate responsibility among these parties. Although relevant regulations emphasize the “main responsibilities” of platforms, this term remains legally ambiguous and provides limited guidance on establishing causation. When addictive outcomes result from multiple interconnected factors, the traditional tort framework, which relies on measurable harm and linear causation, faces significant challenges in determining the unique contribution of platforms.

### 3.3 Barriers to effective algorithmic enforcement

Regulators often face critical challenges in directly observing the internal decision-making processes of recommendation algorithms. These difficulties arise primarily from conceptual ambiguity and institutional fragmentation that undermine effective oversight (6). Platforms frequently invoke trade secret protections to limit transparency. Even when algorithms are disclosed, the technical expertise required for meaningful evaluation often exceeds the current capacity of regulatory bodies. Recent studies also show that many minors bypass or avoid dedicated youth modes on SFVPs due to limited functionality, often preferring the regular mode despite higher risks (15).

## 4 A legal framework for three-tiered prevention among minors

**Figure 1:**
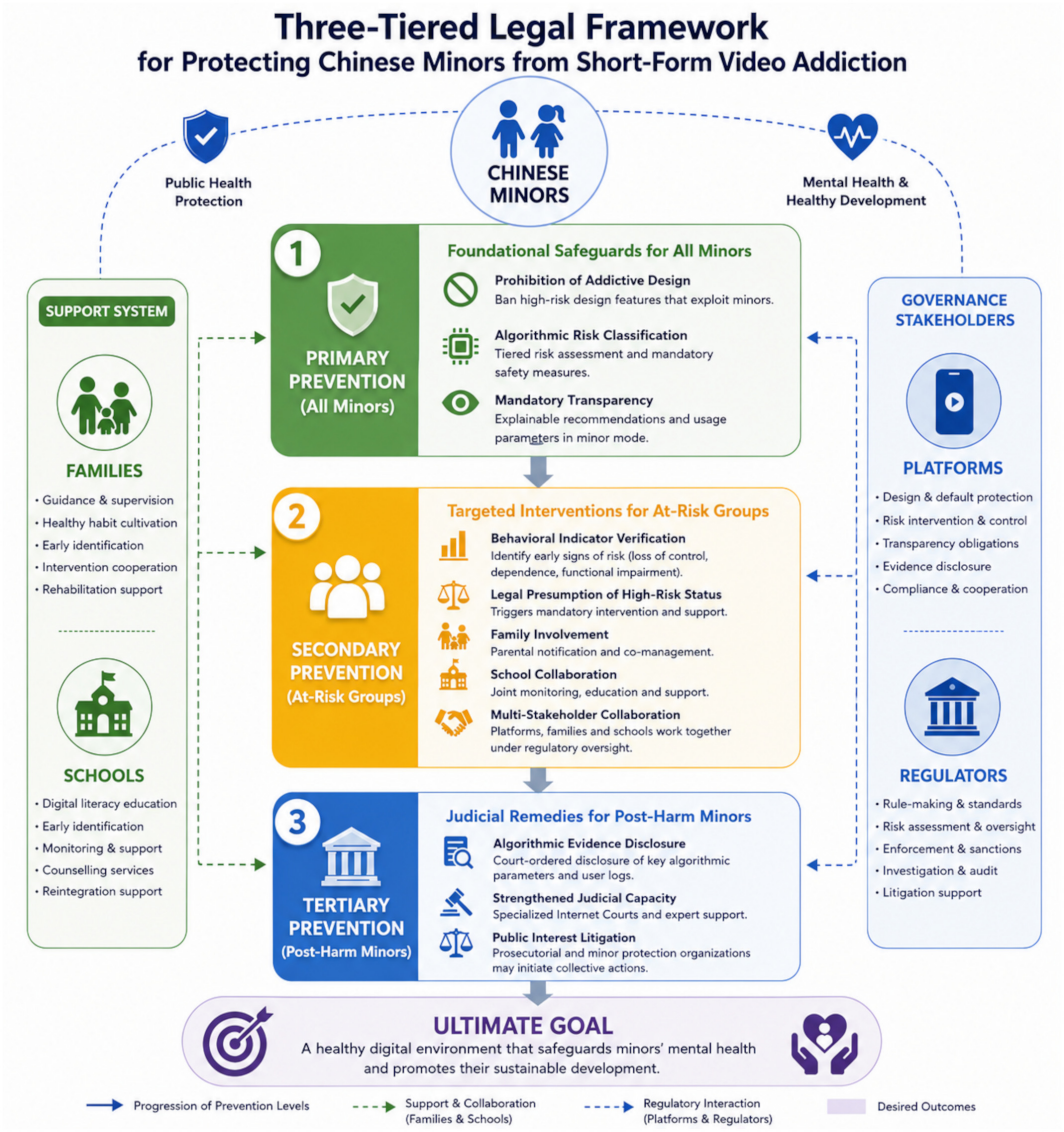
Three-tiered legal framework for protecting Chinese minors from SFVA.

We propose a legal framework drawing on the established public health model of three-tiered prevention (16–19). This tiered approach integrates primary, secondary, and tertiary interventions to act before, during, and after harm occurs. By transforming vague prevention principles into concrete and enforceable governance measures, the framework provides a structured pathway for platform regulation aimed at protecting minors' mental health (See Figure 1).

### 4.1 Primary prevention: foundational safeguards for all minors

The goal of primary prevention is to reduce the overall risk of SFVA among minors by reshaping platform design through legal and technical measures.

Legislation should explicitly prohibit specific high-risk addictive design elements in services targeting minors. These include infinite scrolling, instant feedback reward systems, multi-level task-reward mechanisms, and personalized algorithmic recommendations optimized for maximizing user dwell time. When platforms enable such high-risk features for minors, a legal presumption of “design inducing addiction” should apply. In addition, platforms must activate the highest level of protection mode by default for minor accounts, with any deactivation requiring verifiable parental consent.

Algorithms constitute the core engine of addictive design and therefore require strict oversight. Regulators should establish a tiered algorithmic risk classification system based on measurable “attention capture intensity.” High-risk algorithms must incorporate mandatory safety measures. Furthermore, platforms should provide clear explanations in minor mode regarding why specific content is recommended and which parameters influence usage time. This approach converts abstract algorithmic transparency into a practical, monitorable product feature that empowers users and guardians.

### 4.2 Secondary prevention: targeted interventions for at-risk groups

Secondary prevention focuses on minors showing early indicators of addiction risk and intervenes promptly to prevent progression from high-risk behavior to full addiction.

Effective verification of addiction risk requires behavioral indicators that carry legal consequences, including loss of control, psychological dependence, and functional impairment. For minors, when average daily usage exceeds regulatory threshold and at least one behavioral indicator is confirmed by parents, schools, or mental health professionals, a rebuttable legal presumption of “high-risk status” shall apply. This presumption triggers mandatory interventions and coordinated multi-stakeholder support.

Platforms must fulfill specific obligations toward such high-risk accounts, including more frequent healthy usage reminders, temporary suspension of personalized recommendations, and risk notifications to verified guardians. Regulators should also establish an Algorithmic Ethics Commission, composed of technical experts, legal scholars, and public representatives, to maintain an industry blacklist and impose coordinated sanctions for non-compliance. Complementary measures shall adopt a dual-track approach that combines regulatory governance with digital literacy education, while mobilizing families, schools, and platforms to build a comprehensive protective network for minors (20).

### 4.3 Tertiary prevention: judicial remedies for post-harm minors

Tertiary prevention seeks to mitigate harms that have already occurred and to support recovery through effective judicial remedies.

The current imbalance of information and power between users and platforms remains the primary obstacle for victims seeking justice through litigation. To address this, regulators should introduce an algorithmic disclosure clause empowering courts to order the disclosure of key algorithm parameters and user behavior logs relevant to addiction claims. This provision would shift the burden of proof to platforms, requiring them to demonstrate that their design serves a legitimate, non-manipulative purpose and incorporates adequate safeguards.

Effective judicial remedies also require strengthened institutional capacity. Judicial authorities should leverage existing Internet Courts to develop specialized expertise in algorithm-related cases (21). Furthermore, the authority of prosecutorial bodies and minor protection organizations should be clarified and expanded to initiate civil public interest litigation. For addictive designs that cause widespread social harm, public interest litigation offers a more effective accountability pathway than individual lawsuits alone.

## 5 Limitations and future research

We recognize that short-form video consumption is typically interwoven with other online activities, such as messaging and photo-sharing. Our focused analysis on SFVA is justified by its distinctive algorithmic mechanisms and pronounced cognitive and emotional harms. Nevertheless, we acknowledge that any comprehensive regulatory strategy must ultimately address the broader ecosystem of problematic internet use.

Several practical challenges must be addressed for the proposed three-tiered legal framework to be effectively implemented. Major implementation gaps exist, including a lack of specificity, systematicity, comprehensiveness, and a long-term perspective in current governance approaches (22). China's regulatory bodies currently face documented technical capacity limitations in algorithm evaluation (23). Transitioning from regulatory obligations on paper to effective enforcement will require sustained investment in expertise, infrastructure, and institutional development. Algorithmic transparency and explainability remain another major hurdle. Even when platforms disclose algorithmic parameters, the decision-making processes may not be meaningfully understandable to non-experts. This creates a persistent tension between information accuracy and comprehensibility. Transparency requirements risk resulting in formal disclosure without genuine accountability, while disclosed information may still be manipulated.

Evidentiary uncertainties further complicate judicial application. The burden-shifting mechanism raises unresolved questions regarding the quantum of evidence required to trigger the shift, such as what constitutes prima facie evidence of addictive design and how courts should evaluate competing expert testimony. Regulatory efforts must also carefully balance prevention goals with innovation. Design-based prohibitions and transparency obligations may impose disproportionate compliance costs on smaller platforms. Although the tiered risk approach aims to scale obligations according to potential impact, the risk of over-regulation remains. Excessively detailed rules could lock in outdated solutions and fail to address rapidly evolving addictive mechanisms. Unintended consequences must also be considered, such as strict regulation of mainstream platforms potentially driving minors toward unregulated underground services, or compliance costs disproportionately burdening smaller innovators and reducing diversity in the digital ecosystem.

Future research is essential to strengthen this framework. Priorities include systematic cross-jurisdictional comparisons with recent international developments, such as Australia's *Online Safety Act*, the EU's *Digital Services Act*, and the UK's *Online Safety Act*. Other important areas are comparative effectiveness studies of intervention strategies, with particular focus on behavioral indicator verification and coordinated multi-stakeholder responses. Advancing algorithmic transparency through Explainable AI (XAI) is equally critical; platforms could be encouraged to support XAI research as a condition for operating licenses. Empirical research should also quantify dose-response relationships between platform engagement and functional impairment to establish clear statutory intervention thresholds. Research must further examine how prevention approaches can adapt to emerging technologies such as generative AI, virtual reality, and brain-computer interfaces. Finally, effective policy evaluation measures are needed to monitor both intended effects and unintended consequences in real-world implementation. Concrete practical solutions to overcome the identified challenges, including capacity-building for regulators, graduated evidentiary standards for courts, and proportionate compliance pathways for smaller platforms, will be explored in greater depth in our planned follow-up review article.
